# Improving protein structure prediction using templates and sequence embedding

**DOI:** 10.1093/bioinformatics/btac723

**Published:** 2022-11-10

**Authors:** Fandi Wu, Xiaoyang Jing, Xiao Luo, Jinbo Xu

**Affiliations:** Institute of Computing Technology, Chinese Academy of Sciences, Beijing 626011, China; Toyota Technological Institute at Chicago, Chicago, IL 60637, USA; University of Chinese Academy of Sciences, Beijing 100049, China; Toyota Technological Institute at Chicago, Chicago, IL 60637, USA; Toyota Technological Institute at Chicago, Chicago, IL 60637, USA; Toyota Technological Institute at Chicago, Chicago, IL 60637, USA

## Abstract

**Motivation:**

Protein structure prediction has been greatly improved by deep learning, but the contribution of different information is yet to be fully understood. This article studies the impacts of two kinds of information for structure prediction: template and multiple sequence alignment (MSA) embedding. Templates have been used by some methods before, such as AlphaFold2, RoseTTAFold and RaptorX. AlphaFold2 and RosetTTAFold only used templates detected by HHsearch, which may not perform very well on some targets. In addition, sequence embedding generated by pre-trained protein language models has not been fully explored for structure prediction. In this article, we study the impact of templates (including the number of templates, the template quality and how the templates are generated) on protein structure prediction accuracy, especially when the templates are detected by methods other than HHsearch. We also study the impact of sequence embedding (generated by MSATransformer and ESM-1b) on structure prediction.

**Results:**

We have implemented a deep learning method for protein structure prediction that may take templates and MSA embedding as extra inputs. We study the contribution of templates and MSA embedding to structure prediction accuracy. Our experimental results show that templates can improve structure prediction on 71 of 110 CASP13 (13th Critical Assessment of Structure Prediction) targets and 47 of 91 CASP14 targets, and templates are particularly useful for targets with similar templates. MSA embedding can improve structure prediction on 63 of 91 CASP14 (14th Critical Assessment of Structure Prediction) targets and 87 of 183 CAMEO targets and is particularly useful for proteins with shallow MSAs. When both templates and MSA embedding are used, our method can predict correct folds (TMscore > 0.5) for 16 of 23 CASP14 FM targets and 14 of 18 Continuous Automated Model Evaluation (CAMEO) targets, outperforming RoseTTAFold by 5% and 7%, respectively.

**Availability and implementation:**

Available at https://github.com/xluo233/RaptorXFold.

**Supplementary information:**

Supplementary data are available at *Bioinformatics* online.

## 1 Introduction

Predicting protein structure from its amino acid sequence has been greatly improved by deep learning. Since our proposal (Wang *et al.*, 2017) of the deep ResNet method for protein contact, distance and structure prediction, many research groups have studied similar deep learning methods, such as DMPfold (Greener *et al.*, 2019), tFold (Shen *et al.*, 2021), AlphaFold1 (Senior *et al.*, 2020) and trRosetta (Yang *et al.*, 2020). At the recent 14th Critical Assessment of Structure Prediction (CASP14), DeepMind presented an attention-based method AlphaFold2 (Jumper *et al.*, 2021) that achieved remarkable accuracy. Template-based modeling (TBM), including protein threading and homology modeling, dominated protein structure prediction for many years until the recent application of deep learning to template-free modeling. Our group first employed deep learning to integrate templates, identified by our in-house protein threading method (Wu and Xu, 2021), and co-evolution information to predict inter-residue distance, which is then used to build a tertiary structure. Blindly tested (as a part of the RaptorX server) in CASP13 and CASP14, our method achieved very good performance on the TBM targets (Wu and Xu, 2021). More recent studies (Jumper *et al.*, 2021; Su *et al.*, 2021; Wu and Xu, 2021; Zheng *et al.*, 2019) confirmed that a combination of templates and deep learning may improve tertiary structure prediction for at least TBM targets even if HHsearch (Steinegger *et al.*, 2019) is used to search for templates and build alignments, although HHsearch does not perform as well as our in-house threading software in identifying weakly similar templates. While supervised methods have produced breakthrough results for protein structure prediction, recent studies (Alley *et al.*, 2019; Heinzinger *et al.*, 2019; Rao *et al.*, 2020) have shown that self-supervised protein language models can predict secondary structure and contacts at a reasonable accuracy. DeepMind (Jumper *et al.*, 2021) also integrated multiple sequence alignment (MSA) embedding in AlphaFold2 but did not make use of a protein language model trained by millions of MSAs. The goal of this article is not to develop the best protein structure prediction method. Instead, we study in more detail how templates and MSA embedding generated by protein language models may improve protein structure prediction and its relationship with other factors, such as the number of templates, the difficulty level of a protein target, the depth of its MSA and its sequence length. Specifically, we trained four attention-based models with different feature combinations using the same training set; (i) MSA, (ii) MSA & Template, (iii) MSA & MSATransformer & Template and (iv) MSA & ESM1b & Template and evaluated the model performance.

## 2 Materials and methods

### 2.1 Overview of the method

**Fig. 1.**
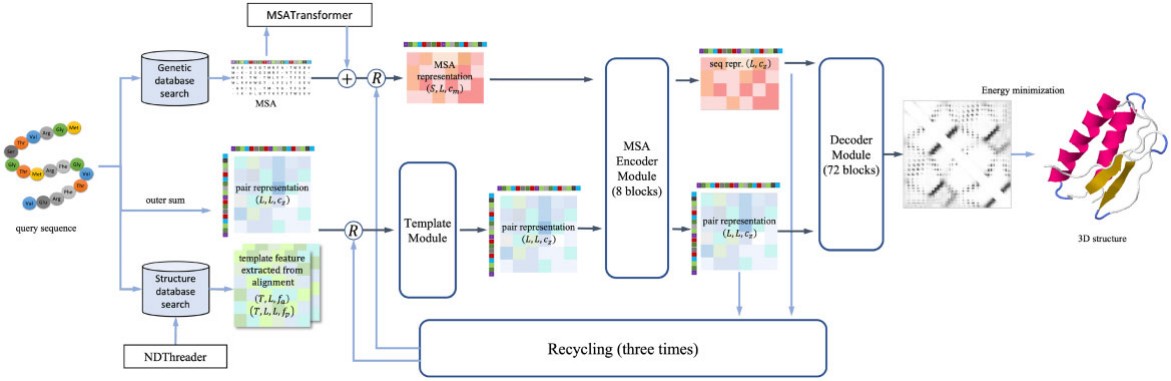
Overview of the network architecture employed in this article. Arrows show the information flow direction. The dark arrows indicate that gradient is used, while the light arrows indicate that gradient is not used

Figure 1 shows our overall network architecture. Given a target protein under prediction, we search genetic databases to build an MSA. We first generate an MSA representation using one-hot encoding, in which individual amino acids, unknown amino acids and gaps are treated as character-level tokens. Then we employ Facebook’s MSATransformer (Rao *et al.*, 2021) to generate the MSA embedding and ESM1b (Rao *et al.*, 2020) to generate the sequence embedding. The details of the embedding module can be found in Supplementary Figure S1. We run NDThreader (Wu and Xu, 2021) for protein threading and select the top five template structures and their alignment to the target. Supplementary Figure S2 shows the template embedding module of our method. The template module embeds template information using three axial attentions (row-wise, column-wise and template-wise attention). This template representation is then concatenated with a pairwise representation using a pointwise attention module. The MSA Encoder module is similar to the RoseTTAFold 2D-track network. We add a pairwise decoder layer of 72 ResNet blocks to predict inter-residue relationships. We also use a recycling strategy similar to AlphaFold2. The detail of our method is presented in Supplementary Section S1. To facilitate the ablation study, we implemented our method so that it is easy to turn on and off a specific module, the template module, the MSA embedding module and the recycling strategy. Our model has 73.3M parameters in total.

### 2.2 Build models from predicted distance and orientation

Our deep model predicts distance distributions for Cb–Cb atom pairs and three types of inter-residue orientation presented in trRosetta (Yang *et al.*, 2020). We discretize the distance into 37 bins: 0–2, 2–2.5, 2.5–3, …, 19.5–20, >20 Å and use one label to indicate an unknown distance when at least one of the two atoms does not have valid 3D coordinates in the PDB file. We discretize the orientation dihedrals *ω*, *θ* and angle *ϕ* into 24, 24 and 12 bins uniformly, respectively.

To build 3D models, we convert predicted distance and orientation distributions into distance potential for tertiary structure prediction by energy minimization. Afterward, we use the fast relaxation protocol in PyRosetta (Chaudhury *et al.*, 2010) for side-chain packing and reducing steric clashes.

### 2.3 Training and validation data

We use the following training and validation protein sets. CathS35 is a representative set of CATH domains (https://www.cathdb.info), in which any two domains share no more than 35% sequence identity. The data are available at ftp://orengoftp.biochem.ucl.ac.uk/cath/releases/dailyrelease/archive/. The version we use for training is v4.2 dated 01/01/2020 and has 32 511 entries. We excluded very short domains and those with too many (>50%) missing Cb and Cb atoms. The protein domains in Cath S35 are shorter than the protein chains in BC40, so it may reduce GPU memory consumption and speed up training by using Cath S35.

PDB clusters all protein chains by MMseq2 (Steinegger and Söding, 2017) at 30%, 40%, …, 95% and 100% sequence identity each week to remove redundancy. BC40 contains 38 774 clusters, and it is a dataset with a 40% cutoff such that the proteins share no more than 40% sequence identity. In total, there are 90 363 protein chains as of January 1, 2020 used for training. While in training, we randomly pick one protein from each cluster.

We excluded very short domains (<25 AAs) and those with too many (>50%) missing Ca and Cb atoms and split them into two non-overlapping subsets: one for training and the other for validation. In the training stage, we choose random *k* templates out of the restricted set of *n* templates, while *k* = Uniform[1, *n*].

### 2.4 Independent test data

We evaluate our model using three test sets: CASP13, CASP14 and CAMEO.

1. CASP13 data. Considering that CASP13 targets are released in 2018, we do not evaluate our model trained using BC40 data (model using Template and MSA embedding generated by MSATransformer). Similar to what we have done in Xu *et al.* (2021), we use TMalign to calculate test target-training protein structure similarity, which is defined as the highest structure similarity (measured by TMscore) between a specific target and all our training and validation proteins. Only T0951-D1, T0971-D1, T1006-D1, T1016-D1 and T1018-D1 have target-training structure similarities larger than 0.9 in CathS35. T0951-D1 is similar to 3w06, T0971-D1 is similar to 3ebt, T1006-D1 is similar to 6g5e, T1016-D1 is similar to 6e4b and T1018-D1 is similar to 3mvi. Only 6g5e and 6e4b were deposited to PDB after 2018, so we excluded these two targets (T1006-D1 and T1016-D1) from the test set. In total, for the CASP13 test set, we have 32 FM targets, 13 FM/TBM targets, 22 TBMHard targets and 43 TBMEasy targets.
2. CASP14 data. We use all officially defined domains with publicly available experimental structures. There are 23 FM targets, 14 FM/TBM targets, 28 TBMHard targets and 26 TBMEasy targets on the CASP14 test set. All targets are released after 2020.
3. CAMEO data. We selected 183 targets released between December 11, 2021 and March 5, 2022 and excluded very short domains (<25 AAs) and those too many (>50%) missing Ca and Cb atoms. In total, there are 88 easy, 77 medium and 18 hard targets.

### 2.5 MSA generation

For each protein domain in CathS35 and BC40, we generated its MSA by running HHblits with E-value = 0.001 on the Uniclust30 (Mirdita *et al.*, 2017) library dated 2017.

For test proteins in CASP13, we run DeepMSA (Zhang *et al.*, 2020) (with default parameters) against the sequence databases Uniclust30 as of October 2017, UniRef90 (Suzek *et al.*, 2015) as of March 2018 and Metaclust50 (Steinegger and Söding, 2018) as of January 2018 to build its MSA.

For CASP14 and CAMEO targets, we search the Uniclust30 as of Aug.2018, UniRef90 as of January 2020, Metaclust50 as of December 2018 and BFD (Steinegger *et al.*, 2019) using HHblits (Remmert *et al.*, 2012) and Jackhmmer (Johnson *et al.*, 2010) to build its MSA.

### 2.6 Template search and alignment generation

We use our in-house protein threading software NDThreader to select the top five templates. During training, we use TMalign to find up to eight top templates for each target and then use NDThreader to generate their alignments to the target. While in training, we randomly select a subset of the eight templates.

### 2.7 Input features

We use the following input features:

- **seq_feat.** It is a one-hot encoding with shape [L, 21] where ‘21’ represents 20 amino acids and one ‘unknown’ amino acid.
- **residue_index.** It is a feature of size [L] consisting of the residue index.
- **msa_feat.** It is a one-hot encoding with shape [S, L, 22], where ‘22’ indicates 20 amino acids + unknown + gap.
- **template_seq_feat.** This is a feature of size [T, L, 11], constructed by the following features extracted from the query and template profile. (i) Amino acid substitution matrix, we use three amino acid substitution matrices BLOSUM80, BLOSUM62 and BLOSUM45 to handle different similarity levels. (ii) Sequence profile similarity, we calculate this by the inner product of position-specific frequency matrix (PSFM) and position-specific scoring matrix (PSSM) in two directions: query PSFM to template PSSM and template PSFM to query PSSM. Both PSFM and PSSM of the test proteins are derived from the profile HHM built by HHblits (Remmert *et al.*, 2012) with E-value = 0.001 and uniclust30 dated October 2017. (iii) Residue mask, which a binary denotes the gap in the template for alignment, which is equal to 1 if and only if the target protein residue has an aligned residue on the template.
- **template_pair_feat.** This is a feature of size [T, L, L, 129] and consists of several pairwise features extracted by a template according to its alignment to a target protein. Three types of Euclidean distance matrices are used for three types of atom pairs: Ca–Ca, Cb–Cb and N–O. We discretize the distance between 3.25 and 50.75 Å uniformly into 38 bins with bin width = 1.25 and use one more bin to represent distance > 50.75 Å. We also use ‘interval distance’, which is calculated by the real distance value minus the lower bound of its bin. We also consider the gap binary matrix for distance. As for the orientation matrix, we employ the inter-residue orientation implemented in trRosetta and calculate the sin and cos values of the orientation angles.

## 3 Results

### 3.1 Accuracy of predicted 3D structures when templates are not used

We have trained a model on the Cath S35 data without using templates and MSA embedding and then evaluated the model quality (measured by TMscore and Global Distance Test (GDT)) of the predicted 3D structures. Tables 1 and 2 and Supplementary Table S2 show that our baseline deep model performs similarly to the PyRosetta version of RosetTTAFold. On the CASP13 test set, the average quality (TMscore) of the first and the best models (selected by energy) obtained by our method is 0.790 and 0.805, respectively while the average quality by RoseTTAFold is 0.796 and 0.809, respectively. On the CASP14 test set, the average quality of the first and best models by our method is 0.746 and 0.760, respectively, slightly better than RoseTTAFold, which obtains 0.744 and 0.758. On the CAMEO test set, our method outperforms RoseTTAFold on Hard, Medium and Easy targets by 0.002, 0.019 and 0.003 in terms of TMscore, respectively. Our method performs slightly worse on large TBMHard and TBMEasy targets, possibly because our training proteins on average are shorter than those used by RoseTTAFold. In terms of the TMscore of the first models, our method outperforms RoseTTAFold on 50 of 110 CASP13 targets, 53 of 91 CASP14 targets and 96 of 183 CAMEO targets, respectively. In terms of the GDT, our method outperforms RoseTTAFold on 48 of 112 CASP13 targets, 52 of 91 CASP14 targets and 93 of 183 CAMEO targets, respectively. The RoseTTAFold’s end-to-end version performs worse than its PyRosetta version.

**Table 1.** The average model quality (measured by TMscore and GDT) on 91 CASP14 targets

CASP14	FM (23)		FM/TBM (14)		TBMHard (28)		TBMEasy (26)	
	TMscore	GDT	TMscore	GDT	TMscore	GDT	TMscore	GDT
RoseTTAFold	0.652/0.661	0.570/0.591	0.736/0.748	0.693/0.713	0.726/0.750	0.657/0.685	0.848/0.859	0.774/0.796
RoseTTAFold + Template	0.645/0.656	0.565/0.584	0.716/0.735	0.668/0.691	0.751/0.762	0.676/0.693	0.872/0.878	0.802/0.816
RoseTTAFold end-to-end	0.6433	0.584	0.72	0.689	0.703	0.622	0.836	0.767
This work	0.643/0.657	0.568/0.589	0.751/0.767	0.713/0.735	0.717/0.735	0.645/0.666	0.861/0.874	0.794/0.814
This work + Template	0.640/0.651	0.561/0.580	0.725/0.744	0.686/0.710	0.802/0.810	0.723/0.739	0.895/0.905	0.845/0.863
This work + Template + seq embedding	0.657/0.667	0.575/0.594	0.765/0.776	0.721/0.736	0.801/0.807	0.724/0.735	0.900/0.906	0.853/0.867
This work + Template + MSA embedding	0.683/0.700	0.610/0.635	0.800/0.812	0.757/0.770	0.800/0.810	0.723/0.740	0.908/0.917	0.865/0.881
This work + Template + MSA embedding (BCData)	0.686/0.703	0.614/0.641	0.801/0.816	0.759/0.777	0.801/0.811	0.723/0.740	0.909/0.917	0.866/0.882
AlphaFold2	0.806	0.764	0.882	0.869	0.845	0.806	0.933	0.915

*Note*: TM represents TMscore and GDT is GDT_TS scaled to [0, 1]. RoseTTAFold means the pyrosetta version, while RoseTTAFold end-to-end means the end-to-end version. BCData means using BC40 as the training set. We use the same MSA as input for all methods. For the method using template information, we use PDB70 released in March 2020 as the template set. For the distance-based method, we use the same folding script to generate 60 decoys and select the best model by energy. The first number in the table means the model selected by energy, while the second number means the best model selected by TMscore. Sequence embedding is generated by ESM-1b and MSA embedding is generated by MSATransformer.

**Table 2.** The average model quality (measured by TMscore, GDT) on 183 CAMEO targets

CAMEO	Hard (18)		Medium (77)		Easy (88)	
	TMscore	GDT	TMscore	GDT	TMscore	GDT
RoseTTAFold	0.574/0.595	0.510/0.527	0.722/0.7415	0.655/0.67	0.857/0.862	0.779/0.789
RoseTTAFold + Template	0.591/0.601	0.527/0.542	0.758/0.768	0.698/0.713	0.889/0.894	0.826/0.836
RoseTTAFold end-to-end	0.54	0.473	0.701	0.633	0.829	0.752
This work	0.576/0.589	0.502/0.518	0.741/0.755	0.671/0.688	0.860/0.864	0.789/0.797
This work + Template	0.631/0.648	0.560/0.578	0.754/0.764	0.705/0.717	0.917/0.921	0.879/0.886
This work + Template + seq embedding	0.622/0.640	0.550/0.574	0.755/0.768	0.709/0.720	0.912/0.915	0.873/0.882
This work + Template + MSA embedding	0.631/0.648	0.560/0.582	0.762/0.771	0.716/0.729	0.910/0.914	0.871/0.880
This work + Template + MSA embedding (BCData)	0.634/0.670	0.577/0.605	0.764/0.777	0.717/0.738	0.916/0.924	0.879/0.890
AlphaFold2	0.773	0.737	0.839	0.829	0.927	0.907

*Note*: TM denotes TMscore and GDT is GDT_TS scaled to [0, 1]. Easy and Hard targets are defined according to the average lDDT of the corresponding structural model. RoseTTAFold means the pyrosetta version while RoseTTAFold end-to-end means the end-to-end version. BCData means using BC40 as the training set. We use the same MSA as input for all methods. For the method using template information, we use PDB70 released in March 2020 as the template set. For the distance-based method, we use the same folding script to generate 60 decoys and select the model by energy. The first number in the table means the first model selected by energy while the second number means the best model selected by TMscore. Sequence embedding is generated by ESM-1b and MSA embedding is generated by MSATransformer.

In our method, we recycle the output pairwise representation of the decoder and the first row MSA representation of the MSA Encoder module. These two types of representations are passed through LayerNorm to update the output of the embedding module. To evaluate the contribution of recycling, we trained another deep model on the same training set but disabling the recycling module. As shown in Supplementary Table S3, the recycling strategy can improve contact prediction significantly. The *t*-test shows that the difference is statistically significant (*P* = 2E−8). This is because the recycling strategy makes the network deeper and brings better MSA and pairwise representation for the MSA Encoder without increasing the number of parameters (Jumper *et al.*, 2021).

### 3.2 Accuracy of predicted 3D structures when templates are used

When templates are used, we have trained one model without MSA embedding and the other with MSA embedding on the Cath S35 data. As shown in Tables 1 and 2 and Supplementary Table S2, on the FM targets, our deep model with templates performs similarly to our deep method without templates. On the FM/TBM targets, our deep method with templates performs slightly worse than when templates are not used. In particular, our method with templates performs particularly badly on two targets T0953s2-D1 and T1047s2-D1 that is because our threading method did not find a similar template for those targets. On the TBMEasy and TBMHard targets, our method with templates performs significantly better (3–11% better in terms of TMscore) than when templates are not used. Most targets in the CAMEO test set have good templates (TMscore >0.6), our model with templates outperformed the model without templates by 4.8% and 9%, respectively, in terms of TMscore and GDT.

**Fig. 2.**
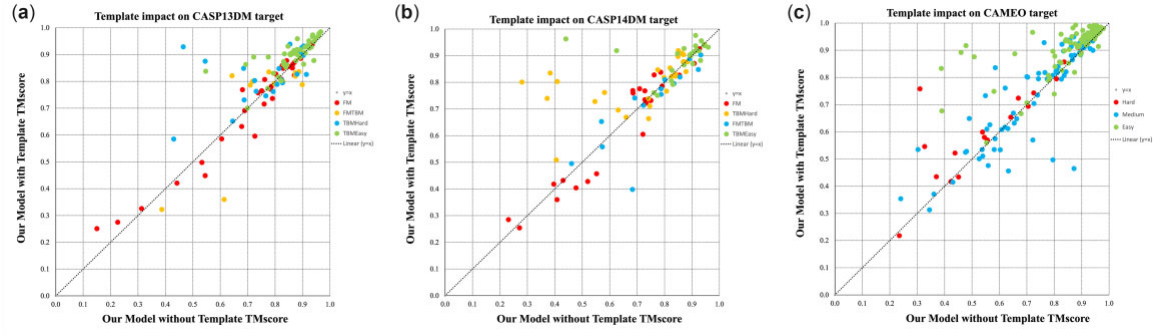
The head-to-head comparison between templates used and not used on the CASP13, CASP14 and CAMEO test sets. The first model is evaluated by TMscore. Different types of proteins are identified in different colors. (**a**) The CASP13 targets; (**b**) the CASP14 targets; (**c**) the CAMEO targets

We also run RoseTTAFold with templates and study their impact. We use the same MSA as input and search the template set released in March 2020 for all methods to make the comparison fair. RoseTTAFold uses HHsearch to find templates. Considering that RoseTTAFold only provides the template set released on March 3, 2020, we only tested RoseTTAFold with templates on the CASP14 and CAMEO test sets. Tables 1 and 2 show that template information generated by HHsearch can improve the model quality of the CASP14 TBMHard and TBMEasy targets and all CAMEO targets but degrade the model quality of the CASP14-FM and CASP14-FM/TBM targets. Figure 2 shows the head-to-head comparison between templates used and not used. Overall template information improves 3D modeling accuracy for 71 of 110 CASP13 targets, 46 of 91 CASP14 targets and all 183 CAMEO targets, respectively. The *t*-test shows that the difference between the model with/without templates is statistically significant (*P* = 3.8E−5 and 1.2E−4 when the first-ranked and the best models are considered, respectively). When templates are used, our method outperforms RoseTTAFold on 54 CASP14 targets (out of 91) and 129 CAMEO targets (out of 183) and underperforms on 36 targets and 52 targets. For CASP14 targets, when the first models are evaluated, our method with templates has an average TMscore and GDT of 0.776 and 0.711, respectively, outperforming RoseTTAFold (0.753 and 0.683, respectively).

### 3.3 Impact of templates on structure prediction

**Fig. 3.**
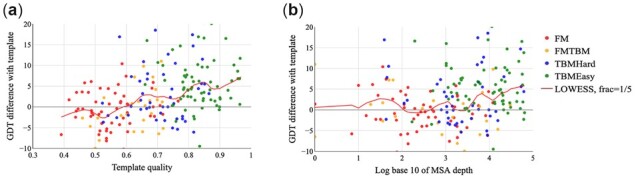
Impact of templates with respect to template quality and MSA depth on the CASP13 and CASP14 test sets. Template quality is defined as target–template structure similarity measured by TMscore. The red lines represent the LOWESS regression curve with hyperparameter frac values of 1/5. Plots are restricted to the range [−10, 20]. Different types of proteins are identified in different colors. *Y*-axis: The difference in GDT computed by the quality of model predicted with template and without template. *X*-axis: (**a**) template quality; (**b**) log base 10 of MSA depth, which is computed by counting the number of sequences in the MSA with a threshold of 90% identity for hhfilter (A color version of this figure appears in the online version of this article)

Figure 3 shows the relationship between MSA depth and template quality (i.e. target–template structure similarity). We evaluate the model with the GDT score on CASP13 and CASP14 test sets. Figure 2a shows that templates improve quality for those targets as long as the target–template similarity (TMscore) is >0.6. Figure 2b shows that for some FM and FM/TBM targets with shallow MSA (MSA depth < 100), templates may still improve the model quality. Our in-house threading software NDThreader can find some templates and generate accurate alignments even for some hard targets (Supplementary Section S4). For proteins with shallow MSA, although template information is not accurate, template information can bring complementary information to shallow MSA.

**Fig. 4.**
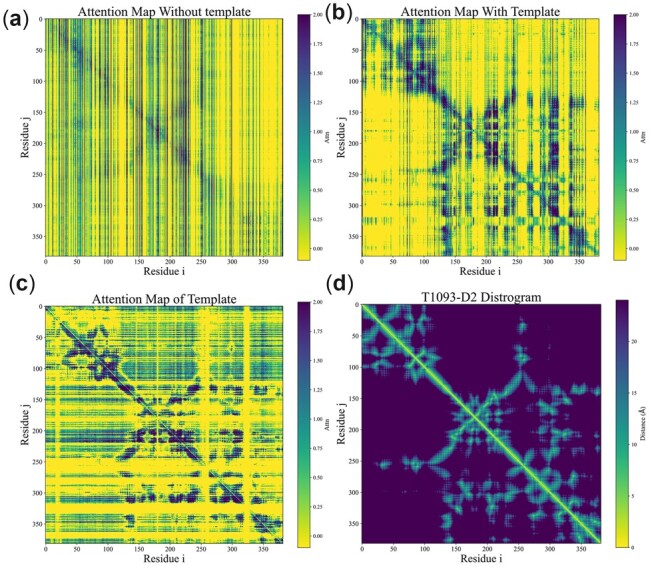
The impact of template information on attention map for T1093-D2. (**a**) Pairwise attention map in the first axial transformer layer without using template information. (**b**) Pairwise attention map in the first axial transformer layer using template information. (**c**) Pointwise attention map of the template. (**d**) T1093-D2 Distrogram

**Fig. 5.**
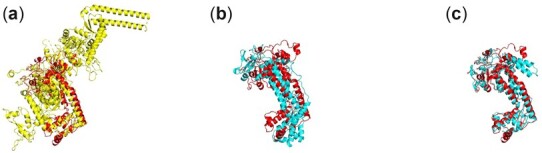
The impact of template information on 3D structure prediction for T1093-D2. Red: native structure. Blue: structure prediction. Yellow: first-ranked template. (**a**) Template 6F6W_D; (**b**) prediction by our method without templates; (**c**) prediction by our method with templates (A color version of this figure appears in the online version of this article)

Figure 4 shows the impact of template information on attention maps for T1093-D2, which is a TBMHard target with 382 residues in CASP14. The template pointwise attention map before MSA Encoder is similar to the distrogram. That is because the template selected by our threading method is similar (TMscore = 0.729 for the first-ranked template) to the target. Figure 3a and b shows the first pairwise attention map with/without template information on the axial transformer layer in the MSA Encoder. Accurate template information brings better initial attention to the MSA Encoder. As shown in Figure 5, template information improves by 0.394 in terms of TMscore for T1093-D2 with high-quality template information.

We also study the impact of the number of templates on structure prediction. As shown in Table 3, templates have little or slightly worse impact on the CASP14 FM and FM/TBM targets. On the TBMHard and TBMEasy targets, when more than five templates are used, the quality of hard targets is worse, while five templates seem to be the best choice for easy targets. On the TBMHard and TBMEasy targets with good templates, once the best template is used, the number of templates has little impact, which shows that our attention-based template module can make use of multiple template information.

**Table 3.** The average model quality (measured by TMscore, GDT) on 110 CASP13 targets with respect to the number of templates

CASP13	FM (32)		FM/TBM (13)		TBMHard (22)		TBMEasy (43)	
	TMscore	GDT	TMscore	GDT	TMscore	GDT	TMscore	GDT
0	0.717	0.659	0.763	0.745	0.765	0.681	0.866	0.811
1	0.725	0.664	0.753	0.733	0.816	0.735	0.887	0.845
5	0.714	0.651	0.746	0.722	0.821	0.740	0.894	0.855
10	0.716	0.655	0.749	0.729	0.816	0.736	0.892	0.852
20	0.711	0.649	0.749	0.729	0.812	0.733	0.890	0.849

*Note*: TM denotes TMscore and GDT is GDT_TS scaled to [0, 1]. Template information was generated by NDThreader against PDB70.

To further study the impact of template quality on the quality of predicted 3D structures, we further use the best templates obtained by the structure alignment tool (TMalign) as input. We run TMsearch (Zhang and Skolnick, 2005) for CASP14 targets (assuming that we have their experimental structures) against the PDB database released in March 2020 and choose the 5 templates with the highest TMscore as the best templates. As shown in Table 4, the TMalign-selected templates significantly improve the prediction accuracy of the FM and TBMHard targets. Supplementary Section S4 shows that NDThreader cannot find a good template (TMscore > 0.5) for almost all CASP14 FM targets and some FM/TBM targets. For the TBMEasy targets, the templates selected by NDThreader are good enough and thus, TMalign-selected templates do not have an advantage over NDThreader.

**Table 4.** The average model quality (measured by TMscore, GDT) on 91 CASP14 targets with respect to the different template features and with or without MSA embedding

CASP14	FM (23)		FM/TBM (14)		TBMHard (28)		TBMEasy (26)	
	TMscore	GDT	TMscore	GDT	TMscore	GDT	TMscore	GDT
NDT	0.640	0.561	0.725	0.686	0.802	0.723	0.895	0.845
TM	0.651	0.571	0.724	0.687	0.826	0.742	0.901	0.852
NDT + emb	0.683	0.610	0.800	0.757	0.800	0.723	0.908	0.865
TM + emb	0.698	0.626	0.812	0.766	0.832	0.753	0.916	0.873

*Note*: emb means using MSA embedding generated by MSATransformer. NDT means using template features generated by NDThreader while TM means using template features generated by TMalign (assuming that we have their experimental structures).

### 3.4 Accuracy of predicted 3D structures when self-supervised MSA embedding is used

Self-supervised methods can learn from billions of sequences and capture extra information not encoded in existing protein structures. When information from self-supervised methods is used, we trained a model with MSA embedding generated by MSATransformer and the other with sequence embedding generated by ESM-1b on the CathS35 set. These models all use template information.

**Fig. 6.**
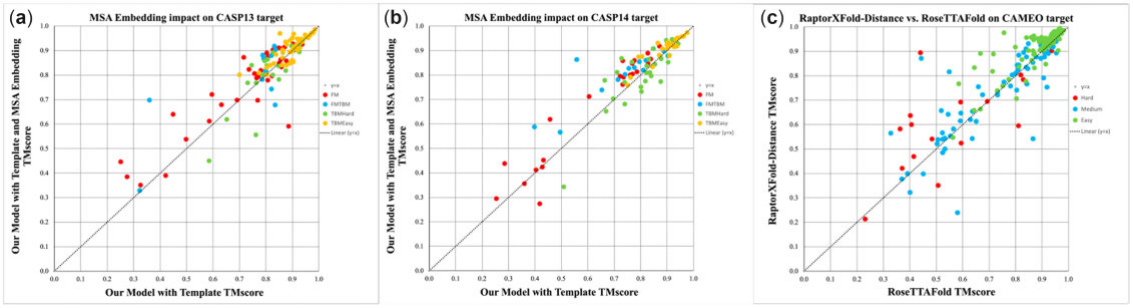
The head-to-head comparison of our method with and without MSA embedding on the CASP13, CASP14 and CAMEO test sets. *Y*-axis: MSA embedding used; *X*-axis: MSA embedding not used. (**a**) CASP13 targets; (**b**) CASP14 targets; (**c**) CAMEO targets

CASP13 FM targets and CASP14 FM targets are challenging since there is no similar template in PDB and most of them have shallow MSAs. When the first models of the CASP13 FM targets and CASP14 FM targets are evaluated, MSA embedding improves model quality by 0.032 and 0.043 TMscore, respectively. Figure 6 shows the head-to-head comparison between our method with and without MSA embedding. On the TBMEasy and TBMHard targets, our method with MSA embedding produces the first models with an average of 0.907 and 0.909 TMscore, respectively. That is, even on the easy targets for which our method with templates shows good performance and generates accurate models, MSA embedding still helps a little. Sequence embedding can improve the model quality of FM and FM/TBM targets, but it is not as significant as MSA embedding. As shown in Table 2, when the first model of the CAMEO targets is evaluated, the model with MSA embedding does not show any advantage in terms of TMscore and GDT, possibly because most CAMEO targets have a similar template (TMscore > 0.6) in PDB.

We also train a model with MSA embedding on BC40 data. BC40 has more long proteins than the CathS35 data. It improves the model quality slightly. Our method with MSA embedding has an average of 0.803 TMscore and 0.741 GDT, respectively, on the CASP14 test set, outperforming RoseTTAFold’s pyrosetta and end-to-end versions by 6% and 10%, in terms of TMscore. Supplementary Figure S4 shows the detail of the comparison between our method and RoseTTAFold on the CASP13, CASP14 and CAMEO set. As a control, single AlphaFold2 predictions have an average of 0.866 TMscore and 0.836 GDT, outperforming our method by a large margin. In terms of TMscore, our method outperforms AlphaFold2 on 11 CASP14 targets (out of 91) and underperforms on 80 targets. AlphaFold2 did much better on the FM and FM/TBM targets. AlphaFold2 combined a number of modules to achieve high-accuracy structure prediction, including end-to-end training from MSA to 3D structure, interactively updating sequence and pairwise representation, using triangular attention to update pairwise representations, the invariant point attention (IPA) module, the recycling strategy, the self-distillation data, etc. We also use AlphaFold2 to predict the structure for the sequences in Uniclust30 (Mirdita *et al.*, 2017) and select those predictions with plddt larger than 80 to build an AlphaFold2-distillation dataset for training. This may improve structure prediction by a 0.04 TMscore on the CASP14 test set. Besides, our current method mainly focuses on distance prediction, we also tried the structure module with IPA to predict coordinates directly but did not observe any improvement, possibly because the scale of training data we used is not big enough and the structural module cannot converge completely under the limited training steps.

### 3.5 Impact of MSA embedding on structure prediction

**Fig. 7.**
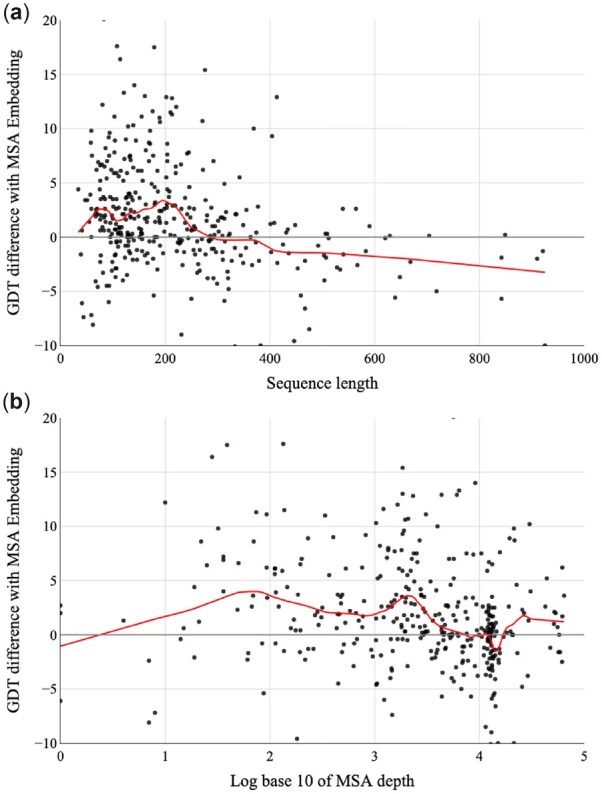
Impact of MSA embedding with respect to sequence length and MSA depth on CASP13, CASP14 and CAMEO test sets. The red lines represent the LOWESS regression curve with hyperparameter frac values of 1/5. Plots are restricted to the range [−10, 20]. *Y*-axis: The difference in GDT computed by the quality of model predicted with and without MSA embedding extracted by MSATransformer. *X*-axis: (**a**) sequence length; (**b**) log base 10 of MSA depth, which is computed by counting the number of sequences in the MSA with a threshold of 90% identity for hhfilter (A color version of this figure appears in the online version of this article)

Figure 7 shows the relationship between MSA embedding, the MSA depth and sequence length. We use the CASP13, CASP14 and CAMEO test sets and evaluate the model with the GDT score. The large impact of MSA embedding on the targets with shallow MSA suggests that MSA embedding is making effective use of unlabeled data on distance prediction. MSA embedding significantly improves the accuracy of short proteins with ≤300 residues. However, for long sequences, MSA embedding has little impact for the following two reasons: (i) the template information is accurate enough for those long sequences; (ii) MSATransformer restricts its training sequence length up to 256 residues.

**Fig. 8.**
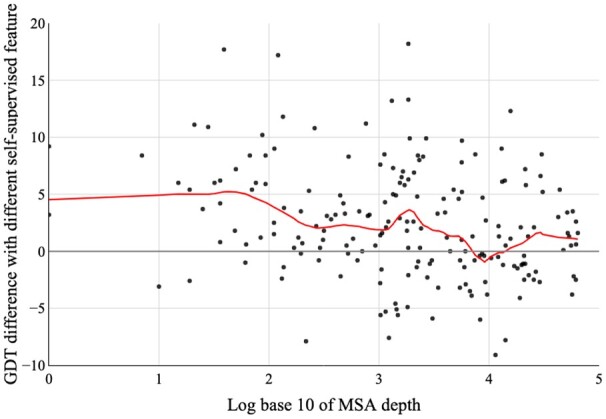
Impact of different self-supervised features with respect to MSA depth on the CASP13 and CASP14 test sets. The red lines represent the LOWESS regression curve with hyperparameter frac values of 1/5. Plots are restricted to the range [−10, 20]. *Y*-axis: The difference in GDT computed by the quality of model predicted with MSA embedding extracted from MSATransformer minus the quality of model predicted with sequence embedding extracted from ESM1b. *X*-axis: log base 10 of MSA depth, which is computed by counting the number of sequences in the MSA with a threshold of 90% identity for hhfilter (A color version of this figure appears in the online version of this article)

We also study the relationship between model quality and different self-supervised features (sequence embedding extracted from ESM1b and MSA embedding extracted from MSATransformer) or MSA depth. As shown in Figure 8, the model with sequence embedding is much worse than the model with MSA embedding when the MSA is shallow (MSA depth < 100). As the MSA depth increases, the quality gap between those two models with different self-supervised features becomes closer, which is because template information is accurate for targets with deep MSA. MSA embedding can bring more information for structure prediction than sequence embedding, which is also stated by Rao *et al.* (2021). We make a runtime analysis for targets with lengths ranging from short to large. Supplementary Figure S3 shows that using the embedding extracted from MSATransformer can achieve considerable improvement with little time overhead.

### 3.6 Better templates can improve AlphaFold2 model quality

**Fig. 9.**
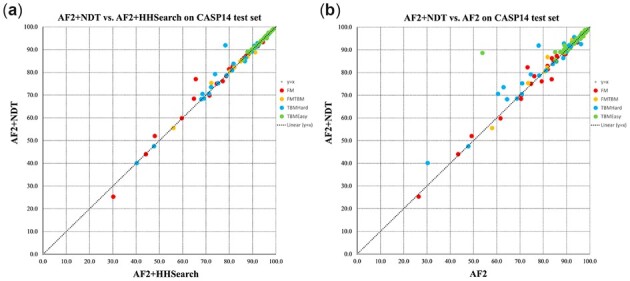
The head-to-head comparison of a single AlphaFold2 model with different template features on the CASP14 set. (**a**) *Y*-axis: use template feature generated by NDThreader; *X*-axis: use template feature generated by HHsearch. They have the same model parameter. (model_1_ptm, trained with template) (**b**) *Y*-axis: use template feature generated by NDThreader (mode_1_ptm, trained with template); *X*-axis: do not use template feature. (model_3_ptm, finetune without template)

Instead of using the templates and alignments generated by HHsearch, we modified AlphaFold2 to use the templates and alignments generated by our in-house threading software NDThreader. Table 5 shows that when better templates generated by NDThreader are used, the model quality improves slightly. With the templates identified by NDThreader, AlphaFold2 may improve model quality by 0.007 and 0.008 on the FM targets and TBMHard targets in terms of TMscore, respectively. The *t*-test shows that the difference is not statistically significant (*P* = 0.045). For the TBMEasy targets, the template information produced by HHsearch is already good and not further improved by using NDThreader. As shown in Figure 9, Good template information can notably improve the predictions of T1060s3-D1, T1038-D1 and T1099-D1 (i.e. TMscore difference > 0.1) without obviously making any protein worse.

**Table 5.** The average accuracy of predicted structures on 91 CASP14 targets

CASP14	FM (23)		FM/TBM (14)		TBMHard (28)		TBMEasy (26)	
	TMscore	GDT	TMscore	GDT	TMscore	GDT	TMscore	GDT
AF2	0.806	0.764	0.882	0.869	0.845	0.806	0.933	0.915
AF2-TPL	0.800	0.758	0.876	0.863	0.848	0.809	0.933	0.915
AF2 + HH	0.800	0.760	0.885	0.873	0.859	0.819	0.947	0.931
AF2 + NDT	0.807	0.766	0.885	0.873	0.867	0.826	0.948	0.932

*Note*: GDT are scaled to [0, 1]. AF2 represents the original AlphaFold2 without using any templates (model_3_ptm). AF2-TPL represents the original AlphaFold2 using all zero-placeholder template features (model_1_ptm). AF2 + HH represents the templates generated by HHsearch (model_1_ptm). AF2 + NDT represents the templates generated by NDThreader. (model_1_ptm). For the AlphaFold2 model, model_1_ptm is trained with templates while model_3_ptm is not.

## 4 Conclusion

In this article, we integrate better template information and MSA embedding extracted from pre-trained protein language models using an attention-based model and study the impact of template information and self-supervised MSA embedding on deep learning-based protein structure prediction. Our test results on the CASP13, CASP14 and CAMEO sets show that both templates and MSA embedding are helpful. We have shown that when a similar template (TMscore > 0.6) is available, our attention-based model can make use of template information and improve structure prediction. The model with templates can improve structure prediction for 52 of 67 CASP14 TBM targets. We found that template information is helpful for targets with shallow MSA (MSA depth < 100). Even for some AlphaFold2-predicted models, better template information can slightly improve model quality.

For the FM targets with shallow MSA, embedding generated by the self-supervised method brings complementary features and improves both distance and structure prediction. Our experimental result shows that MSA embedding can improve structure prediction for 23 of 32 CASP13 FM targets and 20 of 23 CASP14 FM targets.

Overall, the performance of our current model is not comparable with AlphaFold2, but our deep model outperforms RoseTTAFold by 5%, 8% and 5% on the CASP13, CASP14 and CAMEO test sets in terms of GDT score, respectively. Training a deeper model may further improve the performance of our method, we will try this later when computing resources are available.

## Author contributions

J.X. conceived the research. F.W., X.J. and X.L. implemented the algorithms. F.W. carried out the benchmarking experiments. F.W. and X.J analyzed the results. Both F.W. and J.X. wrote the manuscript. All authors revised the manuscript.

## Funding

This work was supported by the National Institutes of Health [R01GM089753 to J.X.]; the National Science Foundation [DBI1564955 to J.X.]; the CSC Scholarship, the NSF of China [under Grants 61925208, 62222214, U22A2028 to F.W], CAS Project for Young Scientists in Basic Research [YSBR-029 to F.W], Youth Innovation Promotion Association CAS and Xplore Prize. The funders had no role in study design, data collection and analysis, decision to publish or preparation of the manuscript.

*Conflict of Interest*: none declared.

## Supplementary Material

btac723_Supplementary_Data

## Data Availability

The RaptorXFold source code is available at https://github.com/xluo233/RaptorXFold.
