# Supplementary material for: Improving protein structure prediction using templates and sequence embedding
Source: Bioinformatics. 2022 Nov 10;39(1):btac723. doi: 10.1093/bioinformatics/btac723 (PMC9805584; doi:10.1093/bioinformatics/btac723)
Supplement: btac723_Supplementary_Data [file btac723_supplementary_data.docx]

Supplementary information for Improving protein structure prediction using templates and sequence embedding

Fandi Wu^1,2,3*^, Xiaoyang Jing^2*^, Xiao Luo^2^, Jinbo Xu^2+^

^1^Institute of Computing Technology, Chinese Academy of Sciences, Beijing, 626011, China, ^2^Toyota Technological Institute at Chicago, Chicago, IL 60637, USA and ^3^University of Chinese Academy of Sciences, Beijing, 100049, China

*These authors contributed equally

^+^Please address all correspondence to [jinboxu@gmail.com](mailto:jinboxu@gmail.com)

# Overview：

In the supplementary, we provide the detail of our methods and results. To begin, we present the features we use in our model in section 1. Secondly, we introduce the algorithm in our model in section 2, including the ThreeAxialTransformer to embed the template information. After that, we compare our method and RosettaFold on the CASP13 test set on section3 and analyze the runtime of our method in section 4. We draw the histogram to present the time costs of different stages. Then, we provide the contribution of the recycling strategy and threading performance of NDThreader [(Wu and Xu, 2021)](https://paperpile.com/c/loWjjZ/SK4a) as a supplement to the main text in section 4-5. In the end, we provide the details of the comparison between our method and RoseTTAFold[(Baek *et al.*, 2021)](https://paperpile.com/c/loWjjZ/kszi) in section 6.

# 1. Model inputs

Table S.1. Input features to the model. Feature dimensions: *N*_res_ is the number of residues, *N*_seq_ is the number of MSA, *N*_templ_ is the number of templates

| **Feature** | **Shape** | **Description** |
| --- | --- | --- |
| f ^seq_feat^ | [*N*_res_, 21] | One-hot encoding of the input acid sequence (20 amino acids and “unknown” amino acids). |
| f ^residue_index^ | [*N*_res_] | Residue index |
| f ^msa_feat^ | [*N*_seq_ , *N*_res_ , 22] | One-hot encoding of the msa (20 amino acids + “unknown” amino acids + gap) |
| f ^template_seq_feat^ | [*N*_templ_ , *N*_res_ , 11] | This is a feature constructed by concatenating the following singleton features extracted from the query and template profile:  “*Template sequence identity*” with shape [*N*_templ_ , *N*_res_ , 1],  “*Amino acid substitution matrix*” with shape [*N*_templ_ , *N*_res_ , 3], “*sequence profile similarity*” with shape [*N*_templ_ , *N*_res_ , 6] and “*residue mask*” with shape [*N*_templ_ , *N*_res_ , 1] |
| f^template_pair_feat^ | [*N*_templ_ , *N*_res_ , *N*_res_ , 129] | This is a feature constructed by concatenating the following pairwise features extracted by a template according to the alignment to its alignment to a target protein: “*template distance matrices*” with shape [*N*_templ_ , *N*_res_ , 41] for three types of atom pairs. “*template orientation matrices*” with shape [*N*_templ_ , *N*_res_ , 2] for three types of inter-residue orientations. |

# 2. Algorithm in our model

In this section, we present the architectural details in Algorithms. We use capitalized operator names when they encapsulate learnt parameters. We use Linear for linear transformation with a weight matrix and bias matrix and use LayerNorm for the layer normalization operating on the channel dimensions with learnable per-channel gains and biases.

We use {z_ij_} to denote all pair representations and {m_si_} for MSA representations.

The inference uses features generated from query sequence, MSA and template information to predict the pairwise distance and orientation in distogram. The model contains 4 recycle iterations, some features generated from the previous iteration will be used to update features in the next iteration. The main steps are described in Algorithm 1, the details will be explained in Algorithm 2 to Algorithm 8.

## **Algorithm 1** RaptorXFold Model Inference

Algorithm 1 shows the inference of our model with recycling iterations. Recycling is found to be helpful in protein structure prediction in AlphaFold2, in our model we recycle the query sequence representation {m_ij_} and pair representations {z_ij_}.

In every recycles iteration, the model converts the sequence feature and MSA feature into MSA representation and pair representation by InputEmbedder (Algorithm 1 line 3), in RecyclingEmbedder (Algorithm 1 line 4) we update the representation from the last recycle iteration. We use the template feature in TemplateEmbedder (Algorithm1 line 5) to update the pair representation. MSAEncoder (Algorithm 1 line 6) produces the final representation. PairwiseDecoder (Algorithm 1 line 7) uses the final representation to predict the pairwise distance and orientation bin.

**def** Inference({f_i_^seq_feat^}, {f_i_^residue_index^}, {f_s, i_^msa_feat^},

{f_ti_^template_seq_feat^}, {f_tij_^template_pair_feat^}, N_cycle_=4):

*# Recycling iteration*

1. m_1i_^prev^, *z*_ij_^prev^ = 0, 0
2. **for all** c ∈ [1, ... , N_cycle_] do

*# MSA embedding*

1. {m_si_}, {z_ij_} = InputEmbedder({f_i_^seq_feat^}, {f_i_^residue_index^}, {f_s, i_^msa_feat^})

*# Inject previous outputs for recycling*

1. {m_1i_}, {z_ij_} = RecyclingEmbedder({m_1i_}, {z_ij_}, {m_1i_^prev^}, {*z*_ij_^prev^})

*# Template embedding*

1. {z_ij_} = TemplateEmbedder({m_si_}, {z_ij_}, {f_ti_^template_seq_feat^}, {f_tij_^template_pair_feat^})

*# MSA Encoder*

1. {m_1i_}, {z_ij_} = MSAEncoder({m_si_}, {z_ij_})

*# Pairwise Decoder*

1. {o_ij_}, {z_ij_} = PairwiseDecoder({m_si_}, {z_ij_})

*# Update data for next recycle*

1. m_1i_^prev^, *z*_ij_^prev^ ← {m_1i_}, {z_ij_}
2. **end for**
3. return {o_ij_}


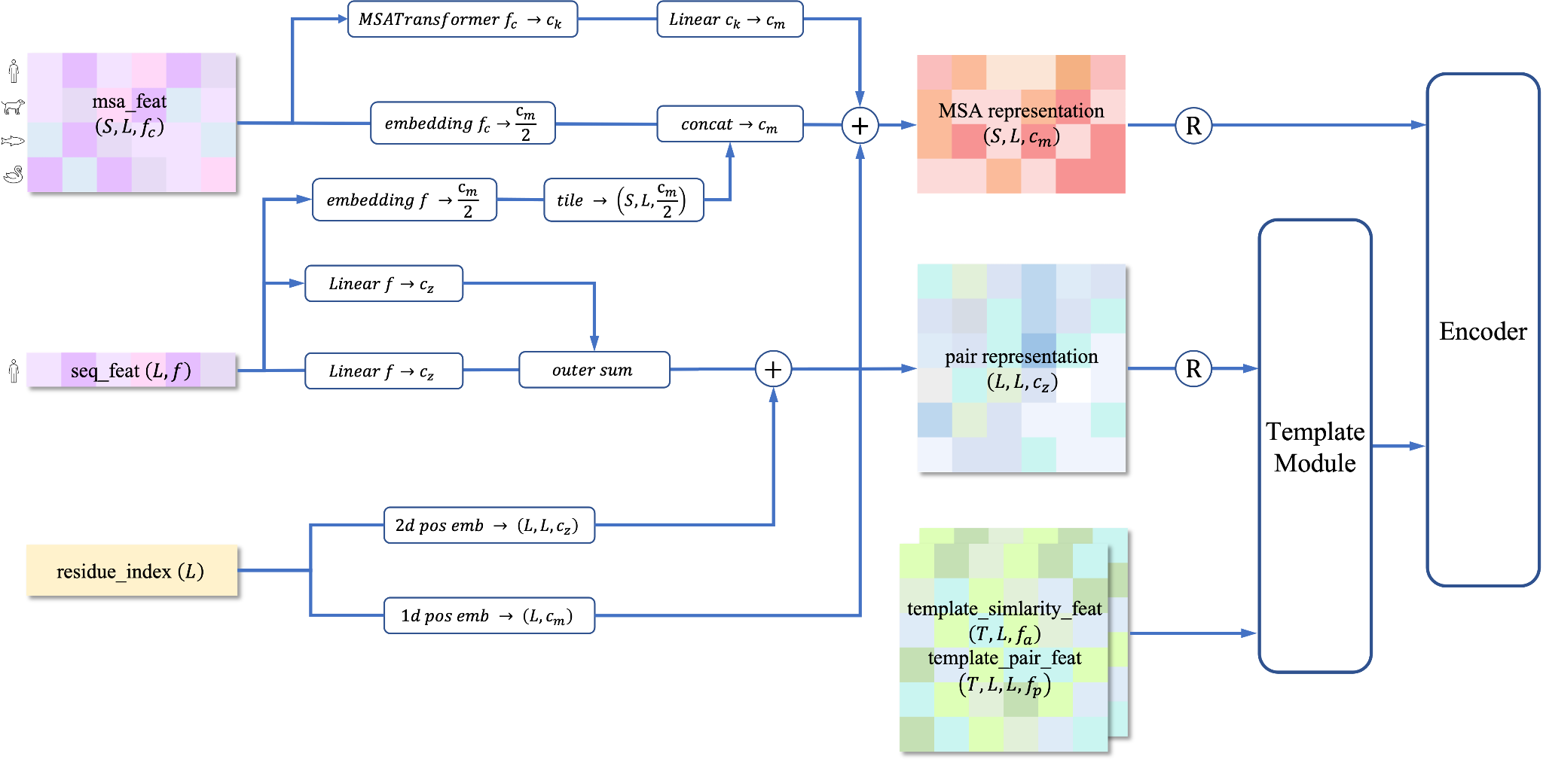


Figure S.1. Input feature embeddings module of RaptorXFold.

## **Algorithm 2** Input embedding using MSATransformer

The sequence feature and MSA feature are converted into MSA representation and pair representation, the details are listed in Algorithm 2 and Algorithm 3. The difference between the two algorithms is that MSATransformer[(Rao, Liu, *et al.*)](https://paperpile.com/c/loWjjZ/5xm3) embedding update MSA representation in Algorithm 2 line 7, ESM_1b[(Rao, Meier, *et al.*)](https://paperpile.com/c/loWjjZ/WkOq) embedding only update representation for query sequence in Algorithm 3 line 7.

**def** InputEmbedder({f_i_^seq_feat^}, {f_i_^residue_index^}, {f_s, i_^msa_feat^}):

1. a_i_ , b_i_ = Linear({f_i_^seq_feat^})
2. z_ij_ = a_i_ + b_j_
3. m_si_ = Embedding({f_s, i_^msa_feat^})
4. c_i_ = Linear({f_i_^seq_feat^})
5. c_si_ = tile(c_i_)
6. m_si_ = concat(m_si_ , c_si_)

#  *Add embedding Generated by MSATransformer*

1. m_si_ += Linear(MSATransformer_Embedding({f_s, i_^msa_feat^}))

*# Add position embedding*

1. z_ij_ += pos_emb_1d({f_i_^residue_index^})
2. m_si_ += pos_emb_2d({f_i_^residue_index^})
3. **return** {m_si_}, {z_ij_}

## **Algorithm 3** Input embedding using ESM-1b

**def** InputEmbedder({f_i_^seq_feat^}, {f_i_^residue_index^}, {f_s, i_^msa_feat^}):

1. a_i_ , b_i_ = Linear({f_i_^seq_feat^})
2. z_ij_ = a_i_ + b_j_
3. m_si_ = Embedding({f_s, i_^msa_feat^})
4. c_i_ = Linear({f_i_^seq_feat^})
5. c_si_ = tile(c_i_)
6. m_si_ = concat(m_si_ , c_si_)

#  *Add embedding Generated by ESM-1b*

1. m_si_ [0]+= Linear(ESM1b_Embedding({f_i_^seq_feat^}))

*# Add position embedding*

1. z_ij_ += pos_emb_1d({f_i_^residue_index^})
2. m_si_ += pos_emb_2d({f_i_^residue_index^})
3. **return** {m_si_}, {z_ij_}

## **Algorithm 4** Embedding of MSA Encoder and decoder outputs for recycling

Feature from the previous iteration is added to the current iteration after layer normalization.

**def** RecyclingEmbedder({m_1i_}, {z_ij_}, {m_1i_^prev^}, {*z*_ij_^prev^}):

1. z_ij_ += LayerNorm(*z*_ij_^prev^)
2. m_1i_ += LayerNorm(m_1i_^prev^)
3. **return** {m_si_}, {z_ij_}


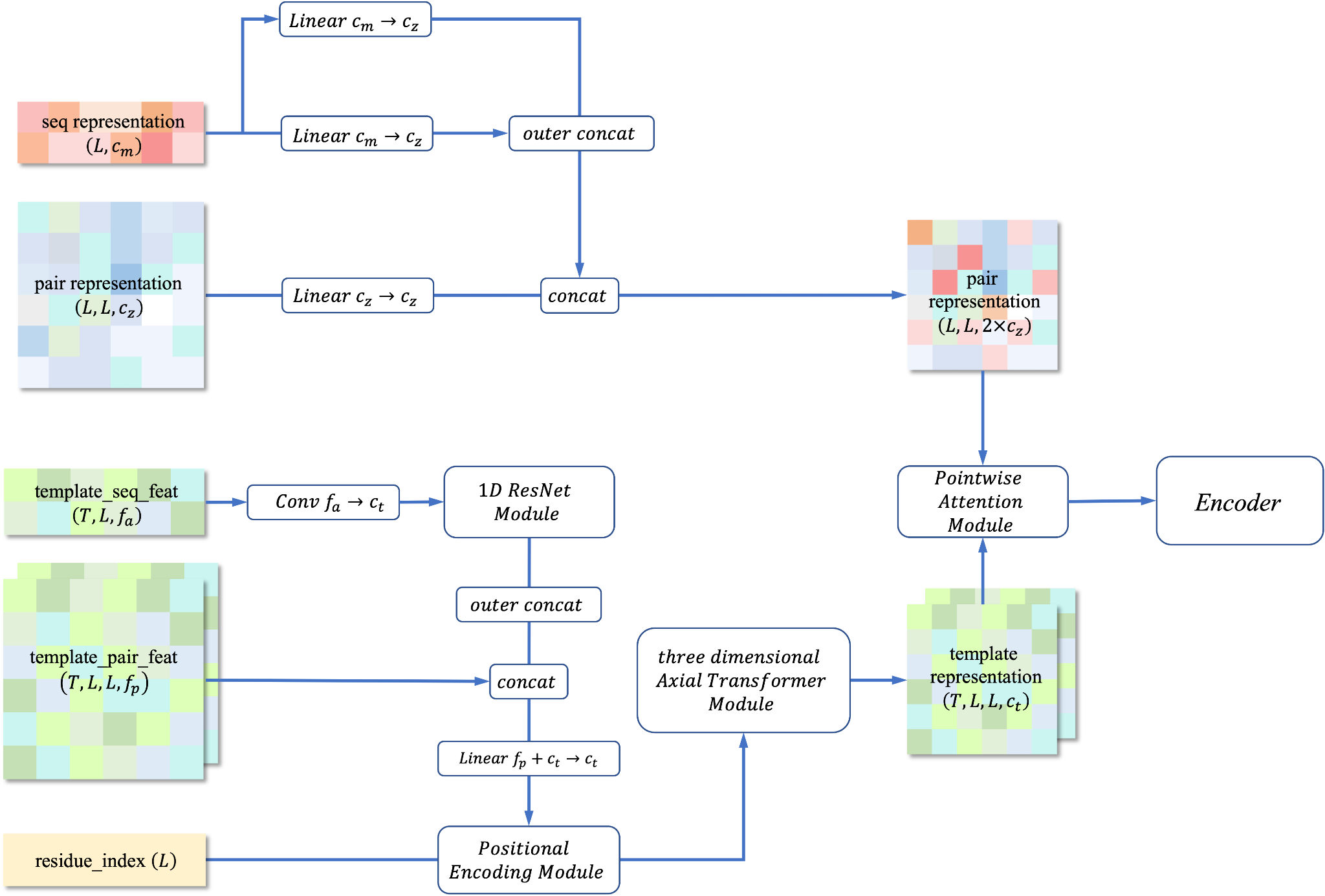


Figure S.2. Template embeddings module of RaptorXFold.

## **Algorithm 5** Template embedding Module

In Algorithm 5 line 5 template singleton features are first passed through a convolution layer then several residue layers. Then the pairwise representation of singleton features is concatenated to the template pairwise feature in Algorithm 5 line 10. The output update itself by the ThreeAxialTransformer layer, then aggregated by Pointwise-Attention, and concatenated with pairwise representation {z_ij_}

**def** TemplateEmbedder({m_i_}, {z_ij_},

{f_ti_^template_seq_feat^}, {f_tij_^template_pair_feat^}, {f_i_^residue_index^}):

*# Sequence embedding*

1. a_i_, b_i_ = Linear({m_i_})
2. m_ij_ = concat(a_i_ , b_j_)
3. {z_ij_} = Linear({z_ij_})
4. {z_ij_} = concat({z_ij_}, {m_ij_})

*# Template embedding*

1. {x_ti_} = convolution({f_ti_^template_seq_feat^})
2. **for all** c ∈ [1, ... , N_block_] do
3. {x_ti_} += ResNetBlock({x_ti_})
4. **end for**
5. y_tij_ = x_ti_ + x_tj_
6. {y_tij_} = concat({y_tij_}, {f_tij_^template_pair_feat^})
7. {y_tij_} = Linear({y_tij_})
8. {y_tij_} = {y_tij_} + pos_emb_2d({f_i_^residue_index^})
9. {y_tij_} = ThreeAxialTransformer({y_tij_})
10. {y_ij_} = PointwiseAttention({y_tij_}, {z_ij_})
11. {z_ij_} = Linear(concat({y_ij_}, {z_ij_})
12. **return** {z_ij_}

## **Algorithm 6** Three Axial Transformer Module

ThreeAxialTransformer contains three multi-head self-attention layers: row-wise, column-wise, and template-wise attention. Row-wise and column-wise attention use every row and column of the pairwise template feature as the query, and template-wise attention uses the whole pairwise template feature as the query to aggregate information across different templates.

**def** ThreeAxialTransformer({z_tij_}, N_block_=2, c=32, N_head_=8):

1. **for all** l ∈ [1, …, N_block_] **do**
2. {z_tij_} ← LayerNorm({z_tij_})

*#*  *Self row-wise Attention*

1. {z_tij_} += MHSelfAttention({z_tij_})
2. {z_tij_} ← LayerNorm({z_tij_})

*#*  *Self column-wise Attention*

1. {z_tij_} += MHSelfAttention({z_tij_}.transpose(1, 2))
2. {z_tij_} ← LayerNorm({z_tij_})

*#*  *Self template-wise Attention*

1. {z_tij_} += MHSelfAttention({z_tij_}.reshape(L*L, T))

*#*  *Feed Forward*

1. {z_tij_} ← LayerNorm({z_tij_})
2. {z_tij_} += FeedForward({z_tij_})
3. **end for**
4. **return** {z_tij_}

## **Algorithm 7** MSA Encoder Module

The MSAEncoder contains 12 blocks. In each block, MSA representation and pairwise representation are used to update each other respectively by MSA2Pair and MHPair2MSA. The implementation is similar to 2D track in RoseTTAFold [(Baek *et al.*, 2021)](https://paperpile.com/c/loWjjZ/kszi).

**def** MSAEncoder({m_si_}, {z_ij_}, N_block_=12):

1. {w_si_} = CalSeqWeight{f_s, i_^msa_feat^}
2. **for all** l ∈ [1, …, N_block_] **do**

*# Transformer Layer*

1. {m_si_} = TransformerLayer({m_si_}, {w_si_})

*# Update pairwise feature using MSA embedding*

1. {z_ij_} = MSA2Pair({m_si_}, {z_ij_})

*# Refine pairwise feature using AxialTransformer Layer*

1. {z_ij_} = AxialTransformerLayer({z_ij_})

*# Update MSA embedding using pairwise feature*

1. {m_si_} = MHPair2MSA({m_si_}, {z_ij_})

*# Feed Forward*

1. {m_si_} ← LayerNorm({m_si_})
2. {z_tij_} += FeedForward({z_tij_})
3. **end for**
4. {m_1i_} = Linear({m_1i_})

*# add query embedding*

1. {z_ij_} += concat({m_1i_}, {m_1j_})
2. **return** {m_si_}, {z_ij_}

## **Algorithm 8** MSA to Pair

In Algorithm 8 line 4, we multiply the MSA representation {a_si_} and sequence weight {w_si_} element-wise. Then in Algorithm 8 line 5, we get the weighted pairwise MSA representation {y_ij_} as the outer product of {a_si_} and {b_si_}. In Algorithm 8 line 9, the {y_ij_} is concatenated to pairwise representation {z_ij_} as the output.

**def** MSA2Pair({m_si_}, {z_ij_}):

1. {m_si_} ← LayerNorm({m_si_})
2. {a_si_}, {b_si_} = Linear({m_i_})

*# Calculate the sequence weight by attention*

1. {w_si_} = CalcSeqWeight({a_si_})
2. {a_si_} = {a_si_} × {w_si_}
3. {y_ij_} = {a_si_} ⨂ {b_si_}
4. {y_ij_} = Linear({y_ij_})
5. {y_ij_} ← LayerNorm({y_ij_})
6. {z_ij_} ← LayerNorm({z_ij_})
7. {z_ij_} = concat({y_ij_}, {z_ij_})

# 3. Evaluation on the CASP13 test set

Table S.2. The average model quality (measured by TMscore and GDT) on 110 CASP13 targets. TM represents TMscore and GDT is GDT_TS scaled to [0, 1]. RoseTTAFold means the pyRosetta version while RoseTTAFold end-to-end means the end-to-end version. We use the same MSA as input for all methods. For the method using template information, we use PDB40 released in March 2018 as the template set. For the distance-based method, we use the same folding script to generate 60 decoys and select the model by energy. The first number in the table means the first model selected by energy while the second number means the best model selected by TMscore. Sequence embedding is generated by ESM-1b and MSA embedding is generated by MSATransformer. We do not test RoseTTAFold with templates due to the lack of template set before CASP13.

|  |  | TM | GDT | TM | GDT | TM | GDT | TM | GDT |
| --- | --- | --- | --- | --- | --- | --- | --- | --- | --- |
| CASP13 |  | FM (32) | | FM/TBM (13) | | TBMHard (22) | | TBMEasy (43) | |
|  | RoseTTAFold | 0.711/0.725 | 0.663/0.687 | 0.767/0.780 | 0.752/0.778 | 0.772/0.789 | 0.691/0.714 | 0.875/0.885 | 0.823/0.844 |
|  | RoseTTAFold end-to-end | 0.6433 | 0.584 | 0.720 | 0.689 | 0.703 | 0.622 | 0.831 | 0.760 |
|  | This Work | 0.717/0.729 | 0.659/0.679 | 0.763/0.788 | 0.745/0.774 | 0.765/0.787 | 0.681/0.708 | 0.866/0.879 | 0.811/0.833 |
|  | This Work + Template | 0.714/0.729 | 0.651/0.676 | 0.746/0.762 | 0.722/0.749 | 0.821/0.829 | 0.740/0.758 | 0.894/0.901 | 0.855/0.870 |
|  | This Work +  Template +  Seq embedding | 0.718/0.730 | 0.662/0.680 | 0.761/0.778 | 0.745/0.766 | 0.808/0.815 | 0.731/0.745 | 0.897/0.902 | 0.856/0.869 |
|  | This Work + Template +  MSA embedding | 0.746/0.761 | 0.695/0.716 | 0.786/0.803 | 0.767/0.787 | 0.816/0.829 | 0.744/0.765 | 0.909/0.915 | 0.874/0.888 |

# 4. Runtime analysis for our method


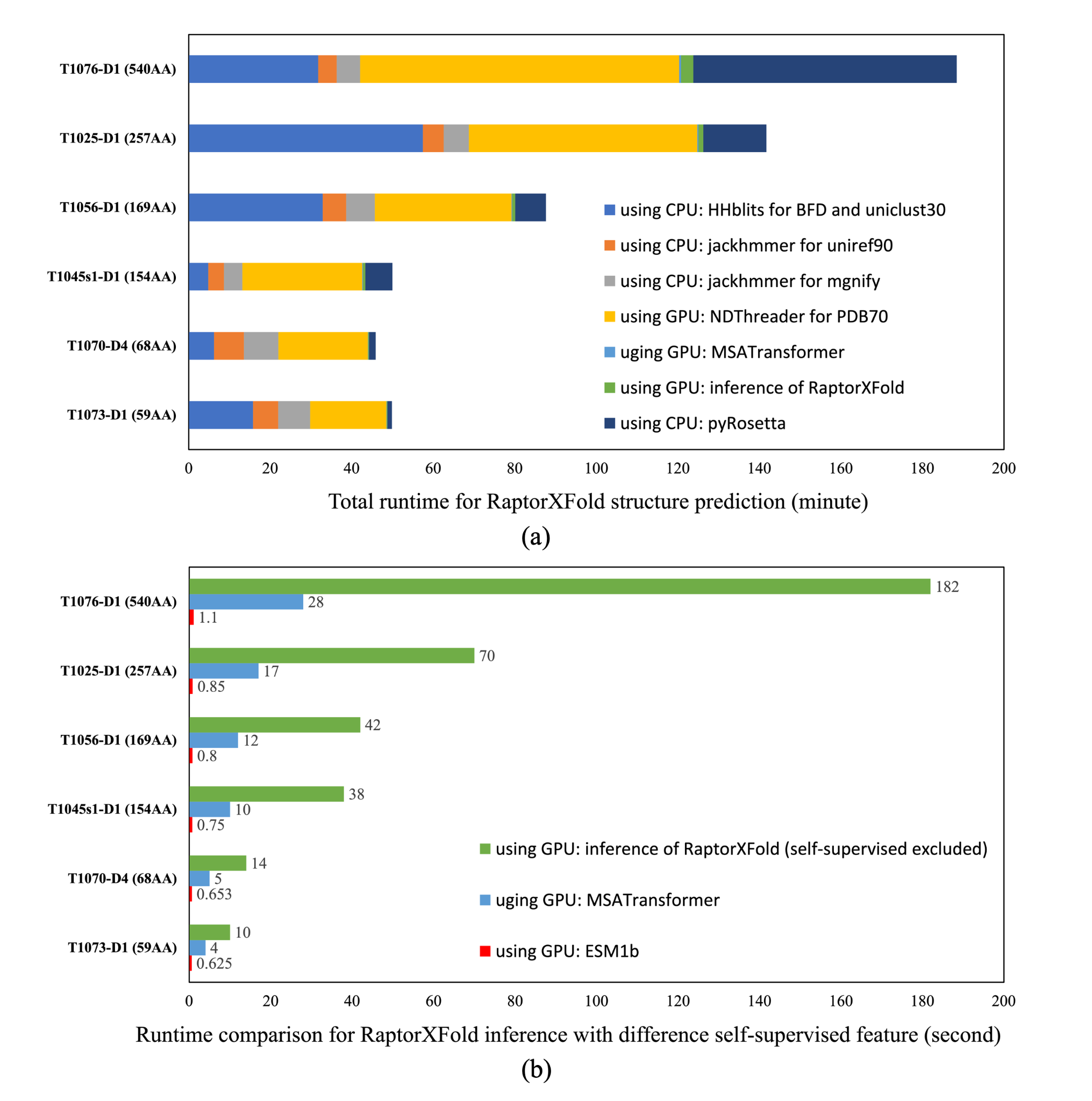


Figure S.3. Runtime analysis for our method for 6 CASP14 proteins on one RTX8000 GPU. The predictions were run entirely on the RTX8000 GPU. However, the HHblits, jackhmmer and pyRosetta stages used only CPUs, and GPUs remained idle in these stages. (a). Total runtime for RaptorXFold structure prediction with template and MSA embedding. MSA embedding was generated by MSATransformer. (b). Runtime comparison for RaptorXFold inference with difference self-supervised feature

To explore the computational overhead on each stage for different proteins, we ran structure prediction for 6 CASP14 proteins ranging from 59 to 540 residues on one RTX8000/48G GPU. We limit the number of CPU cores to 10 and only generate one decoy by pyRosetta. We can use more CPU cores to generate more decoy (e.g., 60 decoys for our performance measures) since that pyRosetta stage can be easier parallelized in multi-CPUs. Figure S.3(a) shows that the runtime of the predictions is up to hours depending on the length of the proteins. NDThreader and HHblits[(Remmert *et al.*, 2011)](https://paperpile.com/c/loWjjZ/OQrO) occupied most of the time on predictions. The NDThreader workflow accounted for 37% of the total runtime for T1073-D1. 47% for T1070-D4, 58% for T1045s1-D1, 38% for T1056-D1, 39% for T1025-D1, 41% for T1076-D1. It takes a long time because NDThreader needs to calculate the score for each target-template pair using deep ResNet on GPU and generate alignment using CRF on CPU. For the MSA embedding generation stage, although MSATransformer has 100M parameters in total, it runs very fast with less than one minute for the longest target, T1076-D1. As shown in Figure S.3, if we use sequence embedding generated by ESM1b, it will be much faster than the MSA embedding generation stage and the time cost will be 1.1 seconds for the longest target, T1076-D1. The longer the protein, the more time proportion it takes for the pyRosetta stage to generate the structure from distance.

# 5. Contribution of Recycling Strategy and Template Information to Contact Prediction

Table S.3. contact prediction precision on the 110 CASP13 targets. TPL denotes using templates as input and REC means using the recycling strategy. We use the same MSA as input for all models.

| CASP13 | | 32 FM targets | | | 13 FM/TBM targets | | | 22 TBMHard targets | | | 43 TBMEasy targets | | |
| --- | --- | --- | --- | --- | --- | --- | --- | --- | --- | --- | --- | --- | --- |
|  |  | Precision of long-range contact prediction | | | | | | | | | | | |
| TPL | REC | Top L | Top L/2 | topL/5 | Top L | Top L/2 | topL/5 | Top L | Top L/2 | topL/5 | Top L | Top L/2 | topL/5 |
| × | × | 60.45 | 70.09 | 83.27 | 70.98 | 79.64 | 88.52 | 70.02 | 79.18 | 88.28 | 82.52 | 90.46 | 95.87 |
| × | √ | 68.79 | 77.25 | 84.91 | 73.29 | 80.51 | 89.23 | 76.9 | 85.34 | 92.5 | 85.46 | 92.29 | 97.56 |
| √ | × | 64.26 | 73.53 | 84.17 | 72.05 | 81.68 | 86.81 | 76.6 | 84.98 | 93.78 | 87.73 | 93.92 | 98.11 |
| √ | √ | 67.81 | 76.69 | 86.65 | 76.46 | 83.71 | 88.99 | 79.21 | 87.53 | 93.58 | 88.99 | 94.38 | 98.59 |

To evaluate the contribution of recycling when model with and without templates, we trained four models on the same Cath S35 and tested the contact prediction precision using the CASP13 test set. Table S.3 shows that the recycling strategy can improve contact prediction significantly.

# 6. Threading performance on CASP13 and CASP14 test set.

Table S.4. Threading performance on the 112 CASP13 targets and 91 CASP14 targets.

| CASP13 | FM (32) | FM/TBM (13) | TBMHard (22) | TBMEasy (45) |
| --- | --- | --- | --- | --- |
| TMsearch with ground truth | 0.584 | 0.653 | 0.764 | 0.844 |
| NDThreader | 0.466 | 0.590 | 0.734 | 0.815 |
| CASP14 | FM (23) | FM/TBM (14) | TBMHard (28) | TBMEasy (26) |
| TMsearch with ground truth | 0.504 | 0.589 | 0.677 | 0.813 |
| NDThreader | 0.367 | 0.531 | 0.647 | 0.817 |

Table S.4 shows the threading performance on the CASP13 and CASP14 test sets. We use TMalign to search the same template database to find structurally the most similar templates for the CASP13 and CASP14 test targets and estimate the difference between the NDThreader alignments and structure alignments. We use MODELLER[(Šali *et al.*, 1995)](https://paperpile.com/c/loWjjZ/LPWi) to build 3D models from NDThreader alignments. We use the target length as the normalization constant in calculating TMscore of a structure alignment. For TBM targets NDThreader alignment is slightly worse than structure alignment, which indicates that NDThreader can generate accurate template information for TBM targets. For most FM and FM/TBM targets, there is still a gap between NDThreader alignment and structure alignment.

# 7. Comparison between RaptorXFold and RoseTTAFold on the CASP13, CASP14 and CAMEO test set


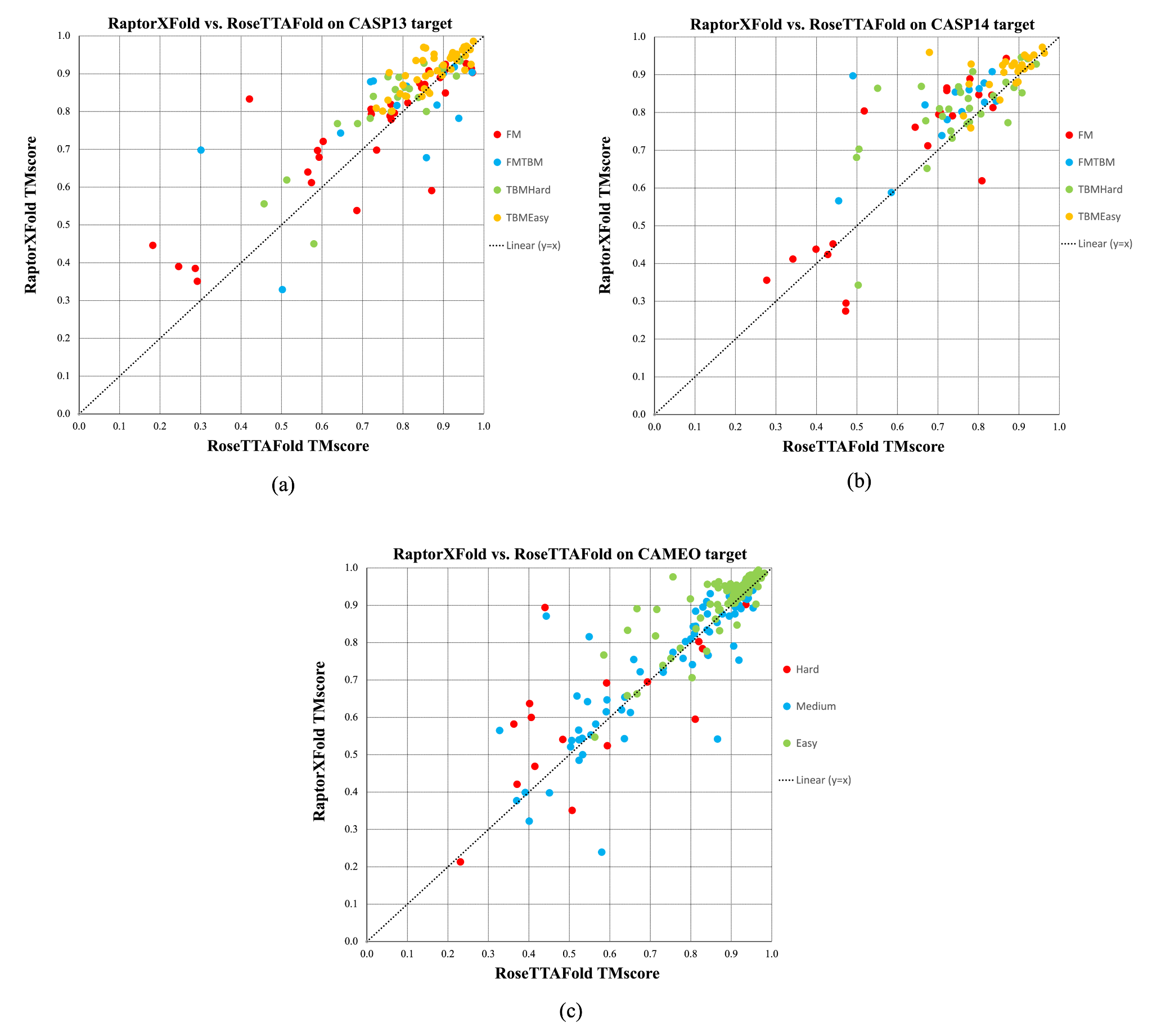


Figure S.4. The head-to-head comparison between RaptorXFold and RoseTTAFold on the CASP13, CASP14 and CAMEO test set. Y-axis: RaptorXFold with template and MSA embedding. X-axis: RoseTTAFold. (a): CASP13 targets. (b): CASP14 targets. (c): CAMEO targets

Figure S.4 shows the comparison between our method and RoseTTAFold on CASP13, CASP14 and CAMEO test sets. Our method was trained with template and MSA embedding on CathS35. The version of RoseTTAFold is v1.0.0, we run the pyRosetta version for testing. To ensure a fair comparison, we use the same MSA as input and search the same template set (released in March 2020 for CASP14 and CAMEO). RoseTTAFold does not provide a template set before May 2018, so we do not use template information for RoseTTAFold for the CASP13 test set. We use energy generated by pyRosetta to select the first-ranked model for comparison.

# Reference

[Baek,M. *et al.* (2021) Accurate prediction of protein structures and interactions using a three-track neural network. *Science*, **373**, 871–876.](http://paperpile.com/b/loWjjZ/kszi)

[Rao,R., Liu,J., *et al.* MSA Transformer.](http://paperpile.com/b/loWjjZ/5xm3) *PMLR*, **139**, 8844-8856.

[Rao,R., Meier,J., *et al.* Transformer protein language models are unsupervised structure learners. *Proc. Natl. Acad. Sci. U.S.A*, **118**, e2016239118.](http://paperpile.com/b/loWjjZ/WkOq)

[Remmert,M. *et al.* (2011) HHblits: lightning-fast iterative protein sequence searching by HMM-HMM alignment. *Nat. Methods*, **9**, 173–175.](http://paperpile.com/b/loWjjZ/OQrO)

[Šali,A. *et al.* (1995) Evaluation of comparative protein modeling by MODELLER. *Proteins: Structure, Function, and Genetics*, **23**, 318–326.](http://paperpile.com/b/loWjjZ/LPWi)

[Wu,F. and Xu,J. (2021) Deep template-based protein structure prediction. *PLoS Comput. Biol.*, **17**, e1008954.](http://paperpile.com/b/loWjjZ/SK4a)
